# Fake news detection on social media: the predictive role of university students’ critical thinking dispositions and new media literacy

**DOI:** 10.1186/s40561-023-00248-8

**Published:** 2023-04-26

**Authors:** Ali Orhan

**Affiliations:** School of Foreign Languages, Zonguldak Bülent Ecevit University, İncirharmanı Campus, Kozlu, Zonguldak, Turkey

**Keywords:** Ability to detect fake news, Critical thinking dispositions, New media literacy, Social media, University students

## Abstract

This study aimed to investigate the predictive role of critical thinking dispositions and new media literacies on the ability to detect fake news on social media. The sample group of the study consisted of 157 university students. Sosu Critical Thinking Dispositions Scale, New Media Literacy Scale, and fake news detection task were employed to gather the data. It was found that university students possess high critical thinking dispositions and new media literacies as well as high fake news detection abilities and there is a positive and moderate relationship among these variables. Also, this study revealed that critical thinking dispositions and new media literacies significantly predicted university students’ abilities to detect fake news on social media and they together explained 18% of the total variance on fake news detection. Besides, university students’ critical thinking dispositions presented a larger effect on their abilities to detect fake news than new media literacies.

## Introduction

With the great enhancement of the internet, social media (SM) has become one of the most widely used sources of information today and a great number of people use SM platforms to learn news (Aldwairi & Alwahedi, 2018). There is a great pile of information on the internet and this can be disseminated very easily and quickly on SM. We can learn breaking news quicker on SM than any other conventional means of communication. However, there is a great problem here. Although SM provides a space for news to spread at an impressive rate, it can also become a hotbed of misinformation (Gaozhao, 2021; Shu et al., 2017). Instead of reaching true and unbiased news, people are bombarded with a great number of fake news (FN) on SM. As a recent example, there has been a rash of digital FN about COVID-19 on SM resulting in undesired health, social, and cultural consequences (Kouzy et al., 2020). Also, FN had an undeniable role in the Brexit Referendum and in the USA Presidential Election in 2016 (Allcott & Gentzkow, 2017; Bastos & Mercea, 2019).

FN can be briefly defined as inaccurate or fictitious content which is released or disseminated as real information although it is not (Gaozhao, 2021). FN—in other words, fabricated news—can be easily disseminated in the form of real news either on SM or through other conventional means of communication (Molina et al., 2021) and it does not have any objective evidence to show the authenticity of the information which it conveys (Pennycook & Rand, 2021). FN attracts a lot more attention and spreads more quickly than real news and it mostly includes emotionally charged language (Vosoughi et al., 2018). Although FN phenomenon has hundreds of years of history (Tandoc et al., 2018) and the ability to detect it has been prized for a long time (Beiler & Kiesler, 2018; Burkhardt, 2017), its reach and mostly deleterious effect have elevated significantly today because of a wide range of SM platforms (Allcott & Gentzkow, 2017). FN is undesirable because it can influence people in a psychological and social way by distorting their beliefs resulting in misinformed and wrong decisions (Zimmermann & Kohring, 2020). Also, FN does not only negatively affect individuals, but it is also harmful to society in many ways. It can have many political, economic, social, and cultural consequences. People can lose their trust in the media (Vaccari & Chadwick, 2020) and balance of the news ecosystem can be ruined because of the increasing spread of FN (Shu et al., 2017).

Therefore, although it is vital to ascertain news veracity on SM where information is easily accessible, it is not easy to do this because of the nature of SM (Hernon, 1995). SM users can have broad access to produce information without any filtering or editorial judgement (Allcott & Gentzkow, 2017) and most of the content on SM is spontaneous and unprofessional (Robinson & DeShano, 2011). Besides, the great volume of information on SM makes it impossible to check the authenticity of the news on SM (Pennycook & Rand, 2019; Zhang & Ghorbani, 2020). Also, people tend to share the information easily and repeatedly with others when they believe FN is true which proliferates the spread of FN (Oh et al., 2018) and they can unwittingly contribute to the dissemination of FN produced by others on SM.

Although producing and consuming FN are clearly two different behaviors, the difference between them has become blurred because of the characteristics of the SM platforms. Individuals can easily create, share, and consume information on SM within a few seconds from anywhere and at any time. Therefore, individuals can easily change their roles from FN producers to consumers, or vice versa (Kim et al., 2021). Also, they can do this intentionally or unintentionally. Therefore, SM is a great place for FN to become extremely influential and spread extremely fast. As SM provides a space for the proliferation of FN and a lot of people rely on SM platforms as the main source of information (Lazer et al., 2018), educating people to fight against FN and equipping them with the necessary tools to identify FN are crucial (Zhang & Ghorbani, 2020). Previous literature indicates that critical thinking (CT) and new media literacy (NML) are two of the most essential tools that can be used by individuals to protect themselves against FN on SM.

### Critical thinking and fake news detection

CT is a functional, reflective, and logical thinking process employed by individuals before deciding what to do or what to believe (Ennis, 2000). In other words, CT can help individuals to make true and reasonable decisions about their actions or on the accuracy of information. CT leads to more accurate and systematic processing of ideas, arguments, and information (Ruggerio, 1988) in which the quality and accuracy of them are examined and evaluated (Lewis & Smith, 1993) and after this careful and logical examination process, they decide to believe or support them. Therefore, it can be said that CT works as armor that protects individuals against fake information (Epstein & Kernberger, 2012). An adequate critical thinker is “habitually inquisitive, well-informed, trustful of reason, open-minded, fair-minded in evaluation, prudent in making judgments, willing to reconsider, diligent in seeking relevant information, reasonable in the selection of criteria, and focused in inquiry” (Facione, 1990, p. 2). For adequate critical thinkers, all assumptions are questionable and divergent ideas are always welcomed. They are always willing to inquire and this inquiry is not affected by their emotions, heuristics, or prejudices, and is not biased in favor of a particular outcome (Kurfiss, 1988).

Therefore, CT can be seen as an effective weapon to combat FN (Bronstein et al., 2019; Wilson, 2018). CT is a thinking process employed by individuals to perceive whether the information is real or fake (Paul, 1990). Good critical thinkers—in other words, people who have high CT skills and dispositions at the same time—tend to examine and evaluate the news they encounter on SM to see if it is accurate and real (Lewis & Smith, 1993). They evaluate the sensibility and accuracy of given news, examine the source of it, and look for sound evidence to trust in the accuracy of it (Mason, 2008). Based on the results of this careful examination process, they can decide whether to share this news with others or not. Therefore, CT can also be seen as an important barrier against the proliferation of FN on SM. While individuals lacking CT tend to share the information easily and repeatedly with others without checking the accuracy of it resulting in the quick spread of FN (Oh et al., 2018), individuals with high CT skills and dispositions tend to evaluate the content and the source of it before sharing. After this careful examination based on sound evidence instead of heuristics and emotions (Kahneman, 2011), they can decide not to share it with others if they decide that it is fake and misleading or it does not have strong arguments, and hence, they can break the chain and decelerate the dissemination of FN on SM. Therefore, it can be said that adequate critical thinkers not only do not fall into traps of FN but also they do not contribute to the dissemination of FN produced by others on SM.

Previous literature has reported some empirical evidence indicating CT has a positive effect on detecting FN on SM. In their study with 1129 participants, Escola-Gascon et al. (2021) found out that CT dispositions significantly predicted the detection of FN. In their experimental study aiming to examine if adding CT recommendations to SM posts can help people to better discriminate true news from FN, Kruijt et al. (2022) concluded that participants who were exposed to CT recommendations presented better performance to detect FN. Lutzke et al. (2019) found that participants exposed to guidelines priming CT performed better to detect FN in their experimental study which was carried out to investigate the effectiveness of guidelines priming CT on willingness to trust, like, and share FN.

### New media literacy and fake news detection

NML can be seen as a broader term which involves different kinds of literacy like classic (e.g., reading and writing), audiovisual (electronic media), digital, and information literacies. It includes some important process skills like access, analysis, evaluation, critique, production, and participation in media content (Hobbs & Jensen, 2009; Lee et al., 2015; Zhang et al., 2014). The interaction between individuals and media content can be divided into two categories, namely, consuming and prosuming (Toffler, 1981). Also, the term of literacy can be divided into two categories which are functional literacies and critical literacies (Buckingham, 2003). While functional literacies, which are related to skills and knowledge, refer to individuals’ capability of knowing how, critical literacies are about individuals’ capability of meaning-making and evaluating the credibility, accuracy, and usefulness of the message (Buckingham, 2003). Based on these two categorizations, a conceptual framework for NML is proposed by Chen et al. (2011). This framework proposes that NML includes functional consuming, functional prosuming, critical consuming, and critical prosuming literacies. While functional consuming literacy is about individuals’ capability of gaining access to created new media content and understanding the message it conveys, critical consuming literacy refers to individuals’ capability of investigating the media content in terms of different perspectives such as cultural, political, social, and economic (Chen et al., 2011). Also, while functional prosuming literacy refers to the ability to create media content, critical prosuming literacy involves the contextual interpretation of the media content by individuals during their activities on media (Chen et al., 2011).

Individuals with high NML are aware of the way the messages are created, disseminated, and commercialized all over the world (Thoman & Jolls, 2004) and can use different media platforms consciously, distinguish and evaluate different media content, investigate the media types, its effects, and the messages they convey in a critical way, and (re)produce new media content (Kellner & Share, 2007). In other words, new media literate individuals can critically access, decode, understand, and analyze the messages which various kinds of media content convey (Leaning, 2017; Potter, 2010) and they can make independent judgements about the veracity of media content (Buckingham, 2015; Leaning, 2017). Therefore, we can say that high NML provides the necessary skills for individuals to actively investigate, evaluate, and analyze the media content and its underlying messages instead of passively consuming the media content and accepting the veracity of the conveying messages which can include potentially misinformation and disinformation without thinking, and hence, it can protect individuals from the negative effects of new media platforms (Hobbs, 2017). New media literate individuals are capable of investigating and evaluating the credibility of the information or news in a critical way, verifying the authenticity of them, and using them ethically. In short, NML increases the possibility that individuals take a critical standpoint toward FN (Kim et al., 2021) and it has an important effect on the degree of consumption and dissemination of FN on SM (Staksrud et al., 2013). Therefore, new media literate individuals can not only protect themselves against the consuming FN but also they are possibly going to be unwilling to share the news without being sure about the accuracy of it, and hence, they are going to have a proactive role in stopping the spread of FN (Parikh & Atrey, 2018).

Previous research has provided some empirical evidence indicating NML has a positive effect on the ability to detect FN on SM. In their study aiming to investigate the relation between students’ level of new media literacies and their ability to discern FN, Luo et al. (2022) concluded that NML and FN detection performance are significantly related to each other. In her experimental study aiming to investigate the effectiveness of media and information literacy on FN detection, Adjin-Tettey (2022) found that media and information literacy trained participants were more likely to detect FN and less likely to share it. Similarly, Al Zou’bi (2022) carried out an experimental study to investigate the effectiveness of media and information literacy on FN detection and concluded that media and information literate students presented better abilities to detect FN. Moore and Hancock (2022) also reported similar results in their experimental study. Besides, Guess et al. (2020) concluded that participants who received media literacy education were more successful in FN detection. Also, Lee et al. (2022) concluded that NML possesses an important role on mitigating the FN problem in their study which was carried out to investigate the effectiveness of NML on perception of FN, media trust, and fact-checking motivation.

### The current study

FN is an important problem and it can pollute the public sphere and harm democracy, journalism, and freedom of expression (Pogue, 2017). Indeed, it is listed as one of the most important threats to society by the World Economic Forum (Del Vicario et al., 2016). Therefore, equipping individuals with the necessary tools to identify FN is crucial (Zhang & Ghorbani, 2020). However, the usage of these necessary tools, especially cognitive ones, to combat FN is not investigated sufficiently (Machete & Turpin, 2020; Wu et al., 2022) and there is a clear need for other studies investigating what can be done and how these tools can be used to combat against FN (Au et al., 2021). Previous literature has showed that CT and NML, which can also be seen as a survival kit for this century, are two of the most essential cognitive tools that can be used by individuals to protect themselves against FN on SM. Although there is a well-established theoretical base regarding the positive effect of CT and NML on FN detection, there is not enough empirical evidence indicating the positive role of CT and NML in fighting against FN in the literature (Xiao et al., 2021; Zanuddin & Shin, 2020). Also, most of the previous research regarding the positive effect of CT and NML on FN detection consists of correlational and experimental studies and there are not enough studies examining the predictive role of CT and NML on FN detection, and hence, empirical evidence regarding the predictive power of these variables is limited. Therefore, we can say that examining the predictive power of CT and NML on FN detection is a promising area of research that may be useful to shed light on what extent these two variables are effective on FN detection on SM. The dearth of research on the predictive role of CT and NML on FN detection provides a sufficient reason for this study aiming to examine the effectiveness of CT and NML on FN detection. Therefore, this study aimed to examine the predictive role of CT dispositions and NML of university students on their ability to detect FN on SM. To this end, the following questions were sought:

1. What are university students’ levels of CT dispositions and NML?
2. Are university students’ Sosu Critical Thinking Dispositions Scale (CTDS) and New Media Literacy Scale (NMLS) scores significant predictors of their ability to detect FN?

## Method

In this non-experimental quantitative study, a cross-sectional survey design was used. University students’ ability to detect FN was determined as the dependent variable of the study and their scores on the CTDS and NMLS were determined as predictor variables.

### Study group

This study was conducted with 157 university students (66 females, 91 males) studying in a state university in Turkey in the academic year of 2022–2023. The students were recruited on a voluntary basis. The mean age of them was 18.96 (SD = 1.00) and their age ranged between 17 and 24. All of the students were in their one-year English preparatory year which is compulsory for them before starting their education in their departments. They are learning only English in this year. The majority of students’ mothers graduated from high school (31.8%) and primary school (29.3%) while most of their fathers are high school (38.2%) and university (25.5%) graduates. A-priori power analysis was conducted using G*Power 3 by Faul et al. (2007) for linear multiple regression analysis (alpha = 0.05; power = 0.95; two predictors) and it showed that the minimal sample size should be 107 to detect a medium effect size (f^2^ = 0.15). So, it can be said that the sample size of 157 was adequate.

### Data collection tools

#### Sosu Critical Thinking Dispositions Scale (CTDS)

The CTDS developed by Sosu (2013) and adapted into Turkish by Orhan (2023) was used to measure the university students’ CT dispositions. The CTDS has 11 items and two sub-dimensions, namely, critical openness (7 items) and reflective skepticism (4 items). Turkish adaptation study with two independent samples indicated that the Turkish version of CTDS has the same factor structure as the original one. The reliability coefficient of the CTDS was found to be 0.92 for sample 1 and 0.94 for sample 2 in the adaptation study. In this study, the reliability coefficient was calculated as 0.80 for the total scale.

#### New Media Literacy Scale (NMLS)

The NMLS developed by Koç and Barut (2016) was used to determine students’ new media literacies. The NMLS has 35 items and four sub-dimensions, namely, functional consumption (7 items), critical consumption (11 items), functional prosumption (7 items), and critical prosumption (10 items). The reliability coefficients of the sub-dimensions ranged between 0.85 and 0.93 while it was calculated as 0.95 for the total scale. In this study, the reliability coefficient was 0.92 for the total scale.

#### Ability to detect fake news

The university students’ ability to detect FN on SM was measured using a FN detection task created by the researcher based on the previous study of Preston et al. (2021). The FN detection task includes six news items, three of them present real news content while three of them include FN content. The FN items include topics related to a claim that Red Cross has ceased its activities in Ukraine (fake), a claim that NASA has stopped its research on oceans (fake), and a claim that Starbucks no more accepts payment by cash money (fake). The first real news item claims that Dwayne Johnson has become the highest-paid actor for the second consecutive year. The second real news item says that an electric bus produced by KARSAN (a Turkish company) started to provide public transportation services in Norway. The third real news indicates that Turkey has become the country that produces the most figs in 2020 with 320 thousand tons of production according to 2020 data from the Food and Agriculture Organization of the United Nations. Two independent and impartial fact-checking websites (www.dogrula.org and www.teyit.org) were used to obtain information related to fake and real news items. These two websites are really popular in Turkey and free to use as a fact-checking resource.

**Fig. 1:**
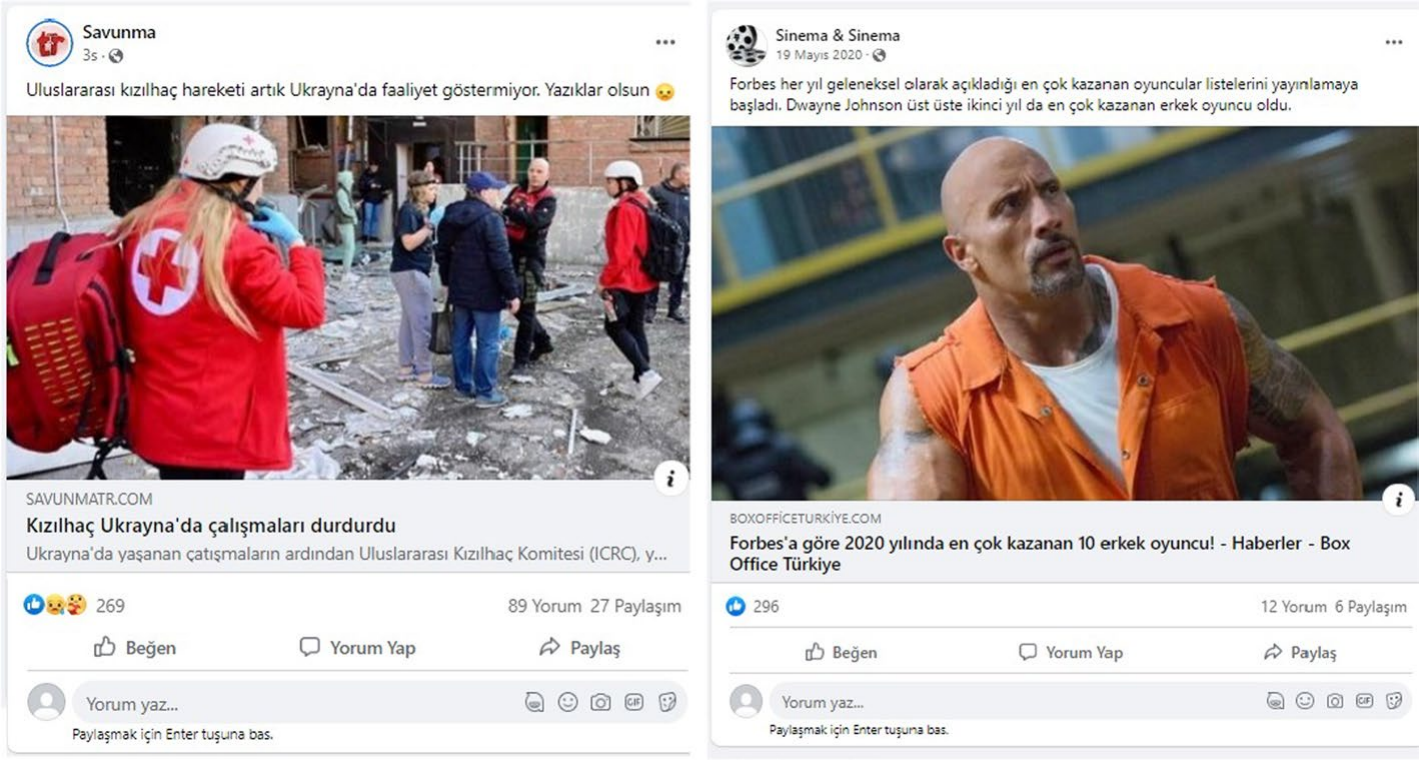
Examples of FN items (on the left) and real news items (on the right)

Four main components, namely, news sharing source, original news item source, content level, and author argument were considered while developing the mock Facebook post items to increase the possibility for the students to evaluate levels of objectivity, professionalism, argument strength, and trustworthiness in the items. The news items look like a typical Facebook news post including likes, comments, and shares in which an article is shared by an organization related to its content (see Fig. 1). For example, the Facebook page named “Sinema & Sinema” is sharing an article from “boxofficeturkiye.com”. The number of comments, likes, and shares are similar between fake and real news items groups in order not to affect students’ choices.

For the first component, more objective content names (e.g. Sinema & Sinema) were used for the real news to influence impressions of professionalism and objectivity while more subjective-sounding content names (e.g. bilim günlüğü) were chosen for FN. Also, for the second component, more objective content suggesting website names were chosen for the real news items (e.g. boxofficeturkiye.com) while FN items included websites suggestive of more subjective content (e.g. savunmatr.com). For the third component, FN items presented short information written in a subjective style and without using any credible source to suggest low trustworthiness. For the fourth component, FN items included author arguments written using emotive language and without references to reliable sources suggesting subjectivity and low levels of argument strength, professionalism, and trustworthiness. On the other hand, I employed the opposite strategies like references to reliable sources and non-emotive language for the real news items.

After preparing the six news items, students were asked to critically analyze each of them and answer four questions prefaced with the text “to what extent do you agree with the following statement”. The first question is “the author and shared article are objective” and it aims to evaluate the level of objectivity. The second question which aims to evaluate professionalism is “the article seems to be produced by a professional”. The third question designed to evaluate the argument strength is “the article presents a strong argument”. The last question is “this source of information is credible and trustworthy” and it aims to evaluate the trustworthiness. Students can answer the questions via 5 point Likert scale ranging from 1 (strongly disagree) to 5 (strongly agree). Students’ responses to the FN are reverse-coded. Scores on the FN detection task can range from 24 to 120 with a midpoint of 60. Higher scores indicate a stronger ability to detect FN while lower scores indicate a weaker ability to detect FN. The reliability coefficient calculated for the FN detection task was 0.84 in this study.

### Data collection

Following the ethical committee approval from ZBEU, the data were gathered in the fall term of 2021–2022 academic year. Privacy and confidentiality issues and the aim of the study were shared with all students and they were informed about their right to withdraw from the study if they want. Students completed the instruments in about 30 min.

### Data analysis

First, all variables were investigated to see whether they had any missing data and it was seen that they had no missing data. After that, the data were investigated in terms of normality and Skewness and Kurtosis values indicated that the data presented normal distribution for each variable (see Table 1). Then, possible multivariate outliers and outliers per variable were checked with Mahalanobis Distance scores and Z transformation values. These scores revealed that the data did not have any influential outliers which should be excluded. Pearson correlation, CI, VIF, and tolerance values were examined to check if there is a high correlation among the variables and it was seen that there is no high correlation. Descriptive statistics, Pearson correlation, and multiple linear regression with enter method were conducted using SPSS 20 statistical software to analyze the data.

**Table 1:** Descriptive statistics for the CTDS, NMLS, and FN detection task and skewness and kurtosis values for each instrument

	Skewness		Kurtosis		$\overline{{\text{X}}}$	sd
	Statistic	Std. Error	Statistic	Std. Error		
CT dispositions	− 0.476	0.194	0.409	0.385	3.89	0.49
New media literacy	− 0.287	0.194	0.095	0.385	3.83	0.49
Ability to detect fake news	0.255	0.194	− 0.528	0.385	76.84	14.52

## Results

As shown in Table 1, university students presented high CT dispositions ($\overline{{\text{X}}}$ = 3.89) and new media literacies ($\overline{{\text{X}}}$ = 3.83). Also, their mean score for the FN detection task is 76.84. As the scores of the FN detection task can range between 24 and 120 with a midpoint of 60, we can say that the students have higher scores than the midpoint indicating that they have high abilities to detect FN.

As shown in Table 2, university students’ CT dispositions (*r* = 0.399) and new media literacies (*r* = 0.303) have a moderate and positive relationship with their abilities to detect FN. Also, there is a positive and moderate relationship between university students’ CT dispositions and new media literacies (*r* = 0.417).

**Table 2:** Inter-correlations among CT dispositions, new media literacies, and FN detection

	New media literacies	Fake news detection
1. CT dispositions	0.417**	0.399**
2. New media literacies	–	0.303**
3. Fake news detection	–	–

**Correlation is significant at p < 0.01

As it can be seen in Table 3, multiple linear regression analysis results indicated that university students’ CT dispositions (β = 0.329, t_(157)=_4.106, p < 0.05) and new media literacies (β = 0.165, t_(157)=_2.062, p < 0.05) significantly predicted their abilities to detect FN on SM (R = 0.426, R^2^ = 0.181, p < 0.01). One-way ANOVA test results showed that the established regression model was significant (F_(2,156)_ = 17.065, p < 0.01). Students’ CT dispositions and new media literacies together explained 18% of the total variance on their abilities to detect FN on SM. Besides, university students’ CT dispositions (β = 0.329) presented a larger effect on their abilities to detect FN than new media literacies (β = 0.165).

**Table 3:** Multiple linear regression results regarding the predictive role of CT dispositions and new media literacies on the ability to detect FN

	B	Std. Error	β	t	p
Constant	20.343	9.926	–	2.049	0.04
CT dispositions	9.687	2.359	0.329	4.106	0.00
New media literacies	4.886	2.370	0.165	2.062	0.04

R = 0.426, R^2^ = 0.181, F_(2,156)_ = 17.065, p < 0.01

## Discussion

This study aimed to investigate the predictive role of CT dispositions and NML of university students on their ability to detect FN on SM. It was seen that university students presented high CT dispositions and NML as well as high abilities to detect FN. Another result obtained in the study revealed that CT dispositions and NML of university students were positively and moderately related to their abilities to detect FN. Also, a positive and moderate relationship between university students’ CT dispositions and NML was found.

This study also revealed that university students’ CT dispositions and new media literacies significantly predicted their abilities to detect FN on SM. Students’ CT dispositions and new media literacies together explained 18% of the total variance on their abilities to detect FN on SM. Previous literature revealed similar results regarding the positive effect of CT dispositions (Escola-Gascon et al., 2021; Kruijt et al., 2022; Lutzke et al., 2019) and NML (Adjin-Tettey, 2022; Al Zou’bi, 2022; Guess et al., 2020; Lee et al., 2022; Luo et al., 2022; Moore & Hancock, 2022) on FN detection on SM. Therefore, we can say that previous literature confirmed the results of this study.

Individuals with high CT skills and dispositions do not make instant decisions about their behaviors or the accuracy of information without employing a systematic and logical thinking process in which they examine and evaluate the quality and accuracy of ideas, arguments, and information (Lewis & Smith, 1993; Ruggerio, 1988). They acquire the most accurate information about their environment and can make the best decision about their actions thanks to CT. Individuals wear CT as armor and protect themselves against fake information (Epstein & Kernberger, 2012). Therefore, we can say that CT is a vital skill for individuals in their daily life and it is even more important during the time they spend on SM where people have been bombarded with a great number of FN. An adequate critical thinker tends to examine and evaluate the accuracy of the news they encounter on SM (Lewis & Smith, 1993) and they are unwilling to share this news with others until they make sure about the sensibility and accuracy of it (Mason, 2008). Therefore, CT not only helps individuals not to fall into traps of FN but also works as a barrier against the dissemination of FN produced by others on SM which makes CT an important and effective weapon to combat FN (Bronstein et al., 2019; Wilson, 2018). Indeed, Machete and Turpin (2020) concluded that previous relevant literature indicated that CT is an important skill to identify FN in their systematic review study aiming to present the current state of the literature on the usage of CT to detect FN.

Individuals with high NML possess the ability to access, decode, understand, and analyze the messages which different kinds of media content convey (Leaning, 2017; Potter, 2010). They can also make independent judgements about the veracity of these media content (Buckingham, 2015; Leaning, 2017). New media literate individuals tend to employ an active process in which they investigate, evaluate, and analyze the media content and its underlying messages instead of passively consuming it because they are aware of the way the media content is created, commercialized, and disseminated all over the world (Thoman & Jolls, 2004) and they know that they can include potentially misinformation and disinformation. Therefore, NML equips individuals with the necessary skills to investigate and evaluate the credibility of the information or news and its source, verify the authenticity of them, and use them ethically. Thanks to NML, individuals take a critical standpoint toward FN (Kim et al., 2021) and it decreases the possibility of consumption and dissemination of FN on SM (Staksrud et al., 2013).

Therefore, we can say that the results of this study indicating CT dispositions and NML were significant predictors of university students’ abilities to detect FN coincide with the theoretical background and the results of previous research. Besides, university students’ CT dispositions presented a larger effect on their abilities to detect FN than new media literacies. This finding shows that CT dispositions are a much more powerful weapon in fighting against FN than NML. CT dispositions equip individuals with the necessary skills to examine and evaluate the quality and accuracy of ideas, claims, and judgments as well as of their source. Also, individuals with high CT dispositions are open to new ideas and they are willing to modify their ideas and arguments when a piece of convincing evidence appears. Therefore, individuals with high CT dispositions are skeptical of any information they encounter in their daily life and they habitually tend to evaluate the veracity of information itself as well as the source of this information. On the other hand, NML is not only about consuming but also producing media content. New media literate individuals are capable of consuming media content in a critical way. They have the necessary skills to actively evaluate the credibility, accuracy, and usefulness of the media content and they can investigate it in terms of different perspectives such as cultural, political, social, and economic. Also, NML is about the capability of gaining access to created new media content, understanding the message it conveys, and (re)producing new media content. They can also use different media platforms consciously, distinguish different media content, and investigate the media types. Correspondingly, we can say that consuming literacies are directly related to FN detection while producing skills have an indirect relation with FN detection ability. On the other hand, CT dispositions are directly related to abilities to detect FN. Therefore, we can say that the result regarding CT dispositions are more effective to combat FN can be explained by this.

In short, this study showed that CT dispositions and NML were significant predictors of university students’ abilities to detect FN. This result which is confirmed by previous research shows the important role of CT dispositions and NML to combat FN which is one of the most important threats to society in today’s world (Del Vicario et al., 2016) and can damage democracy, journalism, and freedom of expression (Pogue, 2017). We can say that individuals possessing high CT dispositions and NML are more competent to combat FN. They can not only protect themselves against FN on SM but also do not contribute to the dissemination of FN by preferring not to share them with other people. Consequently, CT dispositions and NML should be implemented during the effort of fighting against FN on SM. CT dispositions (Kennedy et al., 1991; Lewis & Smith, 1993) and NML (Buckingham, 2003; Hobbs & Jensen, 2009) are teachable skills through appropriate education, and hence, enhancing individuals’ CT dispositions and NML should be included among the most important aims of educational systems because they do not only positively contribute to individuals’ daily and school life (Halpern, 2003; Orhan, 2022a, 2022b; Paul & Elder, 2001) but also work as important barriers against the consumption and dissemination of FN on SM. Therefore, we can say that enhancing individuals’ CT dispositions and NML would be a good idea to equip them with the necessary skills that are useful in the fight against FN on SM. It can be said that this study has significantly contributed to the literature by presenting additional evidence regarding the predictive role of CT dispositions and NML on the ability to detect FN on SM because previous literature lacks enough evidence indicating the predictive roles of these two variables (Xiao et al., 2021; Zanuddin & Shin, 2020). Also, this study differs from the previous studies because it investigated the predictive role of CT dispositions and NML on FN detection comparatively and showed that CT dispositions are a more powerful weapon in fighting against FN than NML.

### Limitations and implications for further research

This study has several limitations although it provides important results regarding the predictive role of CT dispositions and NML on FN detection. First, the study group can be shown as a limitation because this study was conducted with a study group consisting of only university students. Therefore, similar studies can be conducted with other sample groups consisting of students from various educational levels, especially high school because high school students spend most of their time on SM. Second, data collection tools can be shown as another limitation of the study because only self-report quantitative tools were employed to gather the data for this study and these tools can be influenced by social desirability. Therefore, further studies using qualitative or mixed methods can be conducted to better understand the predictive role of CT dispositions and NML on FN detection. Third, several other factors like students’ level of media knowledge may affect their ability to detect FN on SM. However, these factors were not taken into account in this study and this can be shown as the third limitation of the study.

## Data Availability

The datasets used and/or analysed during the current study are available from the corresponding author on reasonable request.

## Abbreviations

CT: Critical thinking
NML: New media literacy
FN: Fake news
SM: Social media
CTDS: Critical Thinking Dispositions Scale
NMLS: New Media Literacy Scale
